# PSTPIP1-associated myeloid-related proteinaemia inflammatory (PAMI) syndrome; a case presenting as a perinatal event with early central nervous system involvement?

**DOI:** 10.1186/s12969-022-00707-5

**Published:** 2022-07-15

**Authors:** Bethany Gillies Whiteside, Hannah Titheradge, Eslam Al-Abadi

**Affiliations:** Birmingham Women’s and Children’s NHS Foundation Trust, Birmingham, UK

**Keywords:** PAMI, PAID, PSTPIP1, PAPA, Autoinflammatory, Perinatal, Fetal distress

## Abstract

**Background:**

We report a three-year-old girl with a potentially unique phenotype of perinatal onset and neurovascular features who was found to have PAMI syndrome. We also compare her case to those previously reported and review the differences between the PSTPIP1-associated inflammatory diseases (PAID) phenotypes and genotypes.

**Case presentation:**

The patient was found to have a heterozygous pathogenic variant in *PSTPIP1* (c.748G > A p.E250K). This variant was shown to be absent in both parents and therefore de novo in the patient.

A literature review was carried out through multiple databases using the terms PSTPIP1, PAID, PAPA syndrome and PAMI syndrome. This information was collected and used to form comparisons between the current literature and our reported case.

**Conclusions:**

Our case contributes to the literature on PAMI syndrome whilst providing an example of a potentially unique clinical phenotype, giving insight into the pre-symptomatic phase of the condition. We highlight the importance of considering PAMI syndrome in the differential for early onset unexplained inflammation. In addition, we explore the possibility that perinatal neurovascular events could be an early feature of PAMI syndrome.

## Background

PSTPIP1 (Proline-Serine-Threonine Phosphatase Interacting Protein 1) is a protein coding gene which regulates multiple cellular functions including T-cell activation, cell migration and interleukin release [1]. Although the underlying pathogenesis is not completely understood, mutations in this gene are thought to result in a spectrum of autoinflammatory disorders characterised by dysregulated interleukin (IL)-1β release and neutrophil responses [2]. Previously, these autoinflammatory syndromes were not well-differentiated from one another. However, as more cases are reported, it is becoming evident that there are multiple genetically and phenotypically distinct conditions. This has led to the recognition of PSTPIP1-associated inflammatory diseases (PAID), encompassing a wide spectrum of clinical phenotypes, many of them associated with unique genetic variants. The most well-recognised of these is PAPA (pyogenic arthritis, pyoderma gangenosum [PG], acne) syndrome; an autosomal dominant autoinflammatory syndrome. The phenotypes of PSTPIP1-associated inflammatory diseases are described in Table 1.

**Table 1:** A summary of the clinical phenotypes of PSTPIP1-associated inflammatory diseases (PAID)

	**Cutaneous inflammation**	**Pyoderma gangrenosum**	**Acne**	**Hepato-splenomegaly**	**Lymphadenopathy**	**Failure to thrive**	**Hidradenitis supportiva**	**Arthritis**	**Ankylosing spondylitis**	**Ulcerative colitis**
Pyogenic arthiritis, pyoderma gangrenosum (PAPA) syndrome	Yes	Yes	Yes	··	··	··	··	Yes	··	··
PSTPIP1-associated myeloid-related proteinaemia inflammatory (PAMI) syndrome	Yes	··	··	Yes	Yes	Yes	··	··	··	··
Pyoderma gangrenosum, acne vulgaris and hidradenitis suppurativa (PASH) syndrome	··	Yes	Yes	··	··	··	Yes	··	··	··
Pyogenic arthritis, pyoderma gangrenosum, acne vulgaris and hidradenitis suppurativa (PAPASH) syndrome	··	Yes	Yes	··	··	··	Yes	Yes	··	··
Pyoderma gangrenosum, acne vulgaris, hidradenitis suppurativa and ankylosing spondylitis (PASS) syndrome	··	Yes	··	··	··	··	Yes	··	Yes	··
Pyoderma gangrenosum, acne vulgaris and ulcerative colitis (PAC) syndrome	··	Yes	Yes	··	··	··	··	··	··	Yes
Psoriatic arthritis, pyoderma gangrenosum, acne vulgaris and hidradenitis suppurativa (PsAPASH) syndrome	··	Yes	Yes	··	··	··	Yes	Yes	··	··

Genovese G, Moltrasio C, Garcovich S, Marzano AV. PAPA spectrum disorders. G Ital Dermatol Venereol. 2020 Jul 2. https://doi.org/10.23736/S0392-0488.20.06629-8

We report a three-year-old girl with a potentially unique phenotype of perinatal onset and neurovascular features who was found to have PAMI syndrome and draw comparisons between her case and that of the current literature.

## Case presentation

A three-year-old girl was first referred to us at nine weeks of age with persistently high inflammatory markers and neutropenia. She was born at full term to two healthy, non-consanguineous parents of African descent. An emergency caesarean section was required for fetal distress and a poor fetal blood gas and meconium-stained liquor was noted at delivery. She required 25 min of supplementary oxygen to maintain her saturations shortly after birth.

At 12 h of age, she was admitted to the neonatal unit for treatment of suspected sepsis. Three hours later, she developed left sided partial seizures for which she was treated with phenobarbitone. Seizures ceased but monitoring continued to show seizure-like activity. Magnetic resonance imaging (MRI) on day four of life showed changes consistent with bilateral infarction and electroencephalogram (EEG) was consistent with multifocal epilepsy. Full body MRI was normal. A decision was made by neurology to hold off commencing regular antiepileptic medication at this point.

Despite several negative blood cultures, a negative meningitis screen and multiple courses of antibiotics, inflammatory markers remained high. C-reactive protein (CRP) fluctuated but never normalised despite the apparent clinical improvement with no further seizures or systemic symptoms. Her feeding, growth and development were normal and examination was otherwise unremarkable. However, at the age of nine weeks, she was persistently anaemic with an ongoing raised CRP. She was therefore referred to the rheumatology clinic with a suspected inflammatory disease and was seen in the autoinflammatory clinic due to her age and presentation with an apparently early onset inflammatory disease.

## Method

Following a referral to our multidisciplinary autoinflammatory clinic, a clinical exome sequencing was carried out. Genomic DNA was extracted from blood leucocytes. The patient underwent clinical exome sequencing using the Agilent SureSelectXT Focused Exome reagent and the Illumina platform. An Autoinflammatory Disease 22-gene panel was applied.

In addition, a literature review was carried out using multiple databases using the terms PSTPIP1, PAID, PAPA syndrome and PAMI syndrome. This information was collected and used to form comparisons between the current literature and our reported case, as summarised in Table 2.

**Table 2:** A summary of PAMI cases within the literature

Author	Number of patients described	Genotype	Phenotype									
			**Hepato-splenomegaly**	**Pyoderma gangrenosum**	**Cystic acne**	**Arthritis**	**Growth failure**	**Other symptoms**	**White cell count (10^9/L)**	**C-reactive protein (mg/L)**	**Serum zinc (umol/L)**	**Serum calprotectin (mg/L)**
Our case (B Gillies Whiteside et al. 2022) [3]	1	E250K	No	No	No	No	No	Seizures secondary to bilateral infarction, speech and cognitive delay	12.1	71	> 160	> 15,000
G Del Borello et al. 2021 [4]	1	E250K	Yes	No	No	No	Yes	Dysmorphic features, developmental delay, lymphadenopathy	Unknown	300	Unknown	Unknown
P Dai et al. 2019 [5]	1	E250K	Yes	Unknown	Unknown	Yes	Unknown	Proteinuria	1.4	> 1000	98	5000
M Mejbri et al. 2019 [6]	1	E250K	Yes	Unknown	Unknown	Yes	Unknown	Multifocal osteomyelitis	Unknown	Unknown	Unknown	Unknown
S Hashmi et al. 2018 [7]	2	E250K	Yes	Unknown	Unknown	Yes	Unknown	Lymphadenopathy	2.1	460	111	Unknown
		E257K	Yes	Unknown	Unknown	Yes	Yes	Lymphadenopathy	1.8	251	143	Unknown
H Klotgen et al. 2018 [8]	1	E250K	Yes	No	Yes	Yes	Unknown	Ulceration, sterile osteomyelitis	Unknown	68	140	2050
E Belelli et al. 2017 [9]	1	E250K	Yes	Unknown	Unknown	Yes	No	Nil	2.8	84	388,000	2.6 × 10^6
E Lindwall et al. 2015 [10]	1	E250K	Yes	Yes	Yes	Yes	Unknown	Osteomyelitis, epistaxis, renal failure	Unknown	65	Unknown	Unknown
K Khatibi et al. 2015 [11]	1	E257K	No	No	No	No	No	Cerebral artery vasculopathy and subarachnoid haemorrhage	Unknown	Unknown	Unknown	Unknown
D Holzinger et al. 2015 [12]	14	E250K	Yes	Yes	No	Yes	No	Dermatitis, liver failure	3.8	146	77	Unknown
		E250K	Splenomegaly	No	No	No	Yes	Nil	3.2	140	118	Unknown
		E250K	Yes	No	No	Yes	Yes	Chronic necrotic lesions, muscular atrophy	3.8	223	147	Unknown
		E250K	Yes	Yes	No	Yes	Yes	Dermatitis, liver failure, impaired motor development	1.5	60	82	Unknown
		E250K	Yes	No	No	Yes	Yes	Chronic necrotic lesions	5	138	113	Unknown
		E250K	Yes	No	No	No	Yes	Abscesses	4.5	206	211	Unknown
		E250K	Yes	No	No	Yes	Yes	IgA nephropathy	1.3	140	200	Unknown
		E250K	No	No	No	No	Unknown	Nil	4	57	98	Unknown
		E250K	No	No	Yes	Yes	Unknown	Abscesses	2.8	24	58	Unknown
		E250K	Splenomegaly	No	No	Yes	Yes	Erythema multiforme, osteitis	1.7	160	52	Unknown
		E250K	Splenomegaly	Yes	No	Yes	No	Ulceration, von Willibrand Factor deficiency	0.08	72	110	Unknown
		E250K	Yes	No	No	No	Yes	Dactylitis, eczema, impaired motor development	1.9	60	144	Unknown
		E250K	Yes	Yes	No	Yes	Yes	Glomerulonephritis	1.6	82	130	Unknown
		E257K	Yes	Yes	Yes	No	No	Recurrent ear infections and hearing loss	1.07	46	64	Unknown
A Demidowich et al. 2012 [13]	5	A230T	Unknown	Yes	Yes	Yes	Unknown	Sterile abscesses	Unknown	Unknown	Unknown	Unknown
		E250Q	Unknown	Yes	No	Yes	Yes	Sterile abscesses, sterile osteomyelitis, recurrent otitis	Unknown	Unknown	Unknown	Unknown
		A230T	Unknown	Yes	Yes	Yes	Unknown	Sterile abscesses, recurrent otitis	Unknown	Unknown	Unknown	Unknown
		A230T	Unknown	Yes	No	Yes	Unknown	Nil	Unknown	Unknown	Unknown	Unknown
		E250K	Yes	Yes	No	Yes	Unknown	Lymphadenopathy	Unknown	Unknown	Unknown	Unknown
H Lee et al. 2012 [14]	2	E257K	Yes	Unknown	Unknown	Unknown	Yes	Osteomyelitis, epistaxis	1.8	250	> 122	Unknown
		E250K	Unknown	Yes	Yes	Yes	Unknown	Ulceration	Unknown	< 5	Unknown	Unknown
B Isidor et al. 2009 [15]	1	Unknown	Yes	Unknown	Unknown	Yes	Yes	Chronic necrotic lesions, recurrent epistaxis	9.9	223	24	2310
T Sugiura et al. 2006 [16]	1	Unknown	Unknown	Yes	No	Yes	Yes	Impaired development	2.4	60	182	12,500
S Fessatou et al. 2005 [17]	1	Unknown	Unknown	Unknown	Unknown	Unknown	Yes	Nil	7	140	4.7	2923
B Sampson et al. 2002 [18]	5	Unknown	Yes	No	No	Yes	Yes	Vasculitis	2	143	200	6500
		Unknown	Yes	No	No	Yes	Yes	Nil	5	200	96	2550
		Unknown	Yes	No	No	Yes	Yes	Nil	1.5	22	200	9000
		Unknown	Yes	No	No	Yes	No	Vasculitis, uveitis, eczema	5	17	175	6100
		Unknown	Yes	No	No	Yes	No	Vasculitis, ulceration	3.8	146	77	1500

## Results

The patient was found to have a heterozygous pathogenic variant in *PSTPIP1* (c.748G > A p.E250K). This variant is absent from population control databases. It has been previously reported in several individuals with a PAMI syndrome, alongside the p.E257K variant [12]. These variants were found solely in patients with the PAMI syndrome phenotype. This variant was shown to be absent in both parents, and therefore is considered to have arisen de novo in the patient.

**Fig. 1:**
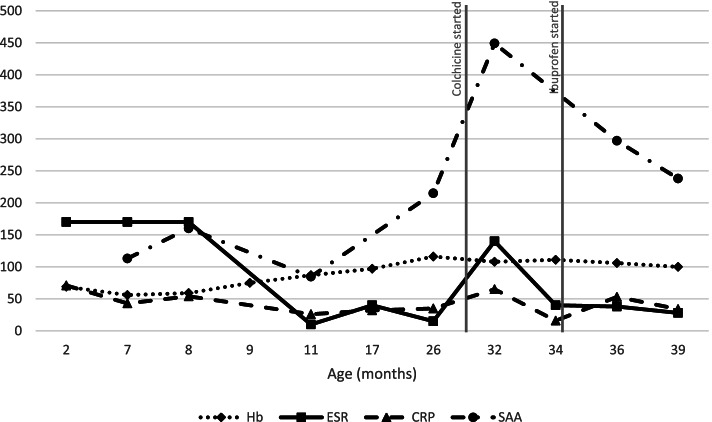
A summary of patient’s blood results. Normal ranges (units): Hb 110–140 (g/l), ESR < 20 (mm/hr), CRP < 5 (mg/l), SAA < 10 (mg/l)

As the patient remains clinically well, her parents are reluctant to commence immunosuppressive treatment and have only allowed a trial course of colchicine followed by ibuprofen, but neither treatment resolved her raised inflammatory markers (Fig. 1). At the age of three years, she shows no clinical features of PAMI, including no fevers, arthritis, hepatosplenomegaly, or lymphadenopathy. Her growth, hearing and vision are normal. Her fine and gross motor development are as expected for her age. However, she has shown speech and cognitive delay and had a single prolonged afebrile seizure requiring treatment with lorazepam. Consistent with the diagnosis, MRP 8/14 and zinc levels were found to be persistently raised at > 15,000 mg/L and > 160 μmol/L respectively. Her raised inflammatory markers (CRP 16-71 mg/l, ESR 10-160 mm/hr) and neutropenia (0.7–2 × 10^9^/l) also persisted (Fig. 1). Repeat MRI showed encephalomalacia and gliosis in keeping with previous infarct [see additional file 1]. EEG was found to be entirely normal.

## Discussion

PSTPIP1-associated myeloid-related proteinaemia inflammatory (PAMI) syndrome, also known as hyperzincaemia and hypercalprotectinaemia, was first described by Sampson in 2002 before the clinical presentation was associated with PSTPIP1 [18]. The syndrome is associated with particularly high levels of myeloid-related protein (MRP) 8 and 14 (approximately 20 times that of PAPA syndrome) [12] which, along with causing an inappropriate inflammatory response, also bind to zinc to cause hyperzincaemia. The zinc is primarily tissue-bound and so this manifests as symptoms of zinc deficiency, including fatigue, failure to thrive and skin, hair and nail involvement [12]. Other clinical features associated with PAMI syndrome include hepatosplenomegaly, lymphadenopathy and pancytopaenia (Table 2) with a significant neutropenia, all of which are usually absent in PAPA syndrome.

PAMI syndrome is associated with the missense variants, p.E250K and p.E257K, which result in a negative charge in the critical coiled-coil domain of the PSTPIP1 protein. This domain is important for pyrin interaction. These substitutions increase the interaction with pyrin, when compared to the p.E250Q variant, associated with PAPA syndrome, although the phosphorylation levels were similar [12]. PAPA syndrome is associated with the variants p.E250Q, p.E256G and p.A230T. The p.E250K and p.E257K missense variants were shown to have G > A amino acid changes at c.748 and c.769 positions respectively, establishing themselves as distinct from the G > C changes seen in p.E250Q.

Our patient did not show any evidence of recurrent fevers, arthritis, skin inflammation or any other previously described symptoms of PAMI syndrome despite the persistently raised inflammatory markers and neutropenia. PAMI syndrome is described as being early onset with an average age of 13 months for first symptoms [12]. It is not clear that the afebrile seizure can be attributed to PAMI syndrome as it has not been previously described. It may be related to the perinatal events; however, it is possible that our patients phenotype could be an antenatal presentation of PAMI. In addition to our patient’s neonatal presentation, Hashmi et al. 2018 report a patient with PAMI syndrome presenting neonatally with pancytopenia at birth [7].

Khatibi et al. 2015 described a patient with PAPA syndrome and an E257K variant with cerebral arterial vasculopathy and a subsequent subarachnoid haemorrhage secondary to a ruptured cerebral artery aneurysm [11]. The authors postulated that this was likely to be secondary to an inflammatory vasculitis due to PAPA syndrome, although based on the mutation reported, it seems likely the patient had PAMI syndrome.

More recently, Del Borrello et al. 2021 described a case of PAMI syndrome and an E250K variant in a young child with significant haematological disease, first manifesting in the neonatal period [4]. The child was also noted to have dysmorphic facial features, global developmental delay and raised inflammatory markers, as well as evidence of diffuse atrophy on brain MRI. Alongside multiple blood transfusions for haemolytic anaemia, the patient was commenced on anakinra and showed dramatic clinical improvement.

Other examples of autoinflammatory diseases manifesting in the neonatal period demonstrate established disease-related damage that suggest the process begins during intrauterine life. For example, in a large UK cohort of cryopyrin-associated periodic syndrome (CAPS), 95% (38 patients) had neurological manifestation, of which 45% (17 patients) had neurological features noticed within the neonatal period, [19] therefore it is not inconceivable that the brain insult may have occurred in utero. Peciuliene et al. 2016 presented the prenatal onset of mevalonate kinase deficiency with fetal hydrops, hepatosplenomegaly and anaemia [20]. Furthermore, Liang et al. (2017) reported prenatal onset of a novel mosaic heterozygous NLRC4 variant in a neonate with congenital anaemia, ascites, and a heavy oedematous placenta with fetal thrombotic vasculopathy, who subsequently developed features of hemophagocytic lymphohistiocytosis (HLH) and died at two months of age [21].

Interestingly, despite novel insights into the causative mutations of the PAMI syndrome phenotype, no consistently effective approach to treatment has been identified. Use of IL-1 inhibitors has arguably demonstrated the best responses but there may also be a role for corticosteroids, cyclosporine or colchicine in symptomatic treatment of this condition [6]. However, few cases are yet to report a resolution of neutropenia in response to treatment.

## Conclusions

Our case provides a unique example of what would have been a mild clinical phenotype if it was not for the possibility that perinatal neurovascular events could be an early feature of PAMI syndrome. In addition, it gives insight into the pre-symptomatic phase of the condition with ongoing elevated inflammatory markers, serum MRP 8/14, serum amyloid and zinc levels as well as ongoing neutropenia. We have highlighted the importance of considering PAMI syndrome in the differential for early onset pancytopenia and we have explored the possibility that perinatal neurovascular events could be an early feature of PAMI syndrome. However, this will require further research in the future in order to make any substantial conclusions. Our case also demonstrates the value of a clinical exome sequencing in investigating early onset phenotypically undifferentiated inflammatory diseases, including during infancy.

## Data Availability

The datasets used and/or analysed during the current study are available from the corresponding author on reasonable request.

## Abbreviations

CAPS: Cryopyrin-associated periodic syndrome
CRP: C-reactive protein
EEG: Electroencephalogram
ESR: Erythrocyte sedimentation rate
HLH: Hemophagocytic lymphohistiocytosis
IL: Interleukin
MRI: Magnetic resonance imaging
MRP: Myeloid-related protein
PAID: PSTPIP1-assocated inflammatory diseases
PAMI: PSTPIP1-associated myeloid-related proteinaemia inflammatory
PAPA: Pyogenic arthritis, pyoderma gangrenosum, acne
PG: Pyoderma gangrenosum
PSTPIP1: Proline-serine-threonine phosphatase interacting protein 1
SAA: Serum amyloid A
