# Correction: PSTPIP1-associated myeloid-related proteinaemia inflammatory (PAMI) syndrome; a case presenting as a perinatal event with early central nervous system involvement?

**DOI:** 10.1186/s12969-023-00797-9

**Published:** 2023-02-06

**Authors:** Bethany Gillies Whiteside, Hannah Titheradge, Eslam Al-Abadi

**Affiliations:** Birmingham Women’s and Children’s NHS Foundation Trust, University of Birmingham, Birmingham, UK

**Correction: Pediatr Rheumatol 20, 49 (2022)**

**https://doi.org/10.1186/s12969-022-00707-5**

The original publication of this article [1] was missing the below affiliation:

- University of Birmingham

The article has been updated to amend this error.
